# Obesity and Overweight Are Independently Associated with Greater Survival in Critically Ill Diabetic Patients: A Retrospective Cohort Study

**DOI:** 10.1155/2021/6681645

**Published:** 2021-02-02

**Authors:** Wentao Huang, Yongsong Chen, Guoshu Yin, Nasui Wang, Chiju Wei, Wencan Xu

**Affiliations:** ^1^Department of Endocrinology and Metabolism, The First Affiliated Hospital of Shantou University Medical College, No. 57 Changping Road, Shantou 515041, China; ^2^Shantou University Medical College, No. 22 Xinling Road, Shantou 515041, China; ^3^Multidisciplinary Research Center, Shantou University, No. 243 Daxue Road, Shantou 515041, China

## Abstract

**Background:**

The relationship between obesity and the outcomes of critically ill diabetic patients is not completely clear. We aimed to assess the effects of obesity and overweight on the outcomes among diabetic patients in the intensive care unit (ICU).

**Methods:**

Critically ill diabetic patients in the ICU were classified into three groups according to their body mass index. The primary outcomes were 30-day and 90-day mortality. ICU and hospital length of stay (LOS) and incidence and duration of mechanical ventilation were also assessed. Cox regression models were developed to evaluate the relationship between obesity and overweight and mortality.

**Results:**

A total of 6108 eligible patients were included. The 30-day and 90-day mortality in the normal weight group were approximately 1.8 times and 1.5 times higher than in the obesity group and overweight group, respectively (*P* < 0.001, respectively). Meanwhile, the ICU (median (IQ): 2.9 (1.7, 5.3) vs. 2.7 (1.6, 4.8) vs. 2.8 (1.8, 5.0)) and hospital (median (IQ): 8.3 (5.4, 14.0) vs. 7.9 (5.1, 13.0) vs. 8.3 (5.3, 13.6)) LOS in the obesity group and overweight group were not longer than in the normal weight group. Compared with normal weight patients, obese patients had significantly higher incidence of mechanical ventilation (58.8% vs. 64.7%, *P* < 0.001) but no longer ventilation duration (median (IQ): 19.3 (7.0, 73.1) vs. 19.0 (6.0, 93.7), *P* = 1). Multivariate Cox regression showed that obese and overweight patients had lower 30-day (HR (95% CI): 0.62 (0.51, 0.75); 0.76 (0.62, 0.92), respectively) and 90-day (HR (95% CI): 0.60 (0.51, 0.70); 0.79 (0.67, 0.93), respectively) mortality risks than normal weight patients.

**Conclusions:**

Obesity and overweight were independently associated with greater survival in critically ill diabetic patients, without increasing the ICU and hospital LOS. Large multicenter prospective studies are needed to confirm our findings and the underlying mechanisms warrant further investigation.

## 1. Introduction

Over the last few decades, the global prevalence of obesity has increased drastically [1]. It is currently one of the biggest public health issues incurring substantial social, economic, and medical burdens [2, 3]. In the general population, obesity has been recognized as a risk factor for early death [3, 4]. However, there is growing evidence that obese patients have a better prognosis than their normal weight counterparts; a phenomenon called the obesity paradox [5–7].

Obese individuals usually have a greater burden of comorbid conditions and are more likely to develop physiologic derangements and critical illness [3, 8]. With the pandemic of obesity, the number of obese patients admitted to the intensive care unit (ICU) has increased [8, 9]. Whether the phenomenon of obesity paradox exists in the population with critical illness has received widespread attention and has been investigated in a series of studies [10–21]. It should be noted that the proportion of diabetic patients recruited in the above studies was limited and that only one study [13] reported the relationship between obesity and hospital mortality in diabetic patients, despite in the context that diabetes mellitus (DM) has become another major global health issue and common comorbidity in the ICU [22, 23]. Therefore, the effect of obesity on critically ill diabetic patients is not completely determined.

A clear understanding of the relationship between obesity and the outcomes of ICU diabetic patients will contribute to better treatment and care of these patients. In this study, we aimed to assess the effect of obesity on the outcomes among critically ill diabetic patients in ICU, hypothesizing that body mass index (BMI) as a surrogate indicating obesity is associated with the prognosis of ICU diabetic patients. The effect of overweight (preobesity) was also assessed.

## 2. Materials and Methods

### 2.1. Database

The data of the present study were from a publicly accessible critical care database named Medical Information Mart for Intensive Care III (MIMIC-III, version 1.4), which is a large, single-center database containing information of 46520 patients who were admitted to various ICUs of the Beth Israel Deaconess Medical Center (BIDMC) between 2001 and 2012. The detailed description of the MIMIC-III database is available elsewhere [24]. After completing the National Institutes of Health web-based training course and the Protecting Human Research Participants examination, we obtained approval to access the database (Certification Number: 8971151). Since the study was an analysis of the third party anonymized publicly available database with preexisting institutional review board (IRB) approval, IRB approval from our institution was exempted.

### 2.2. Study Population

Adult diabetic patients (aged 18 years or above) admitted to the ICU for the first time were included. Patients were excluded if they meet the following criteria: (1) with nontype 1 or nontype 2 DM, or unclassified DM; (2) have been hospitalized in ICU for less than 1 day; and (3) with an unavailable BMI. We also excluded those who were underweight (BMI < 18.5 kg/m^2^) in our analysis since our focus was on obesity and overweight. The final participants were divided into three groups according to the BMI classification criteria set by the World Health Organization (WHO) [25]: normal weight (BMI: 18.5 kg/m^2^ to <25.0 kg/m^2^), overweight (BMI: 25.0 kg/m^2^ to <30.0 kg/m^2^), and obesity (BMI ≥ 30.0 kg/m^2^).

### 2.3. Data Extraction

We executed the data extraction with structure query language (SQL) in Navicat Premium (version 15.0; PremiumSoft CyberTech Ltd.). The following data were extracted: age, gender, ethnicity, admission type, ICU type, the classification of DM, BMI, Acute Physiology Score (APS) III, Sequential Organ Failure Assessment (SOFA) score, and multiple comorbidities. BMI was calculated as body weight (kg)/height (m^2^). Body weight information was obtained from medical documents recorded within 24 hours of admission to ICU while height information was obtained from medical documents recorded during this or other hospitalizations. The SOFA score and APS were calculated on the first day of each patients' ICU stay. The extracted comorbidities included acute myocardial infarction (AMI), stroke, heart failure, renal failure, respiratory failure, and malignancy. In this database, the true ages of patients over 89 years old were obscured and their median age was 91.4 years, so we used this value as a surrogate age for those patients.

### 2.4. Clinical Outcomes

The primary outcomes in our study were 30-day and 90-day mortality, which were defined as death observed within 30 and 90 days after ICU admission. The secondary outcomes included ICU mortality, hospital mortality, ICU length of stay (LOS), hospital LOS, and the requirements for renal replacement therapy (RRT) and vasopressor therapy. The vasopressor included adrenaline, norepinephrine, and dopamine. The incidence and duration of mechanical ventilation during the ICU stay were also assessed.

### 2.5. Statistical Analysis

Statistical analyses were performed by using STATA software (version 16.0; Stata Corp LP, College Station, TX) and SPSS software (version 26.0; IBM, Armonk, NY). The normality of distribution of continuous variables was tested by *Q*-*Q* plots. Continuous variables with normal distribution were described as the mean ± standard deviation (SD) and were compared by one-way ANOVA or Kruskal-Wallis (K-W) test as appropriate. Continuous variables with nonnormal distribution were presented as median and interquartile range, and the K-W test was used for comparisons between groups. Categorical variables were expressed as absolute values and percentages and were compared using chi-square test or Fisher's exact test as appropriate.

Survival analyses were performed with the log-rank test to determine whether BMI category affected 30-day and 90-day mortality. Kaplan-Meier survival curves were generated. We also constructed three Cox regression models to further explore the association between BMI category and 30-day and 90-day mortality. There was no confounder adjustment in the unadjusted model. Model-1 was adjusted for the confounders age, gender, and ethnicity, while model-2 was adjusted for the confounders that were considered clinically relevant or that showed a statistically significant univariate relationship (*P* < 0.20) with the outcomes. The proportional hazards (PH) assumption of the Cox regression model was evaluated by Schoenfeld residual test and time-by-covariate interactions test [19, 26, 27]. When the PH assumption was not satisfied, the effect of the covariate was considered to be time varying; thus, this covariate was modeled as a time-dependent covariate by adding an interaction term computed as the byproduct of time and the individual covariate [ln(time) × covariate] into the model, and then, the extended Cox model was constructed [27–29]. The results of the Cox regression model were presented as hazard ratios (HRs) with 95% confidence intervals (95% CIs).

Additionally, we implemented two post hoc analyses. First, subgroup analyses were conducted to examine whether the effects of obesity and overweight differed across various subgroups, including admission type, ICU type, DM classification, AMI, stroke, heart failure, renal failure, respiratory failure, and malignancy. The Cox regression model was adjusted for the confounders' age, gender, and ethnicity. Second, the included patients were subdivided into five groups according to BMI and Cox regression models were reconstructed to further investigate the association between different degrees of obesity and mortality. The five groups included the normal weight group (BMI: 18.5 kg/m^2^ to <25.0 kg/m^2^), overweight group (BMI: 25.0 kg/m^2^ to <30.0 kg/m^2^), class I obesity group (moderate obesity; BMI: 30.0 kg/m^2^ to <35.0 kg/m^2^), class II obesity group (severe obesity; BMI: 35.0 kg/m^2^ to <40.0 kg/m^2^), and class III obesity group (morbid obesity; BMI ≥ 40.0 kg/m^2^) [25].

All reported *P* values were two sided and were adjusted for multiple comparisons using the Bonferroni correction. A *P* value of less than 0.05 was considered statistically significant.

## 3. Results

### 3.1. Subject Characteristics

**Figure 1:**
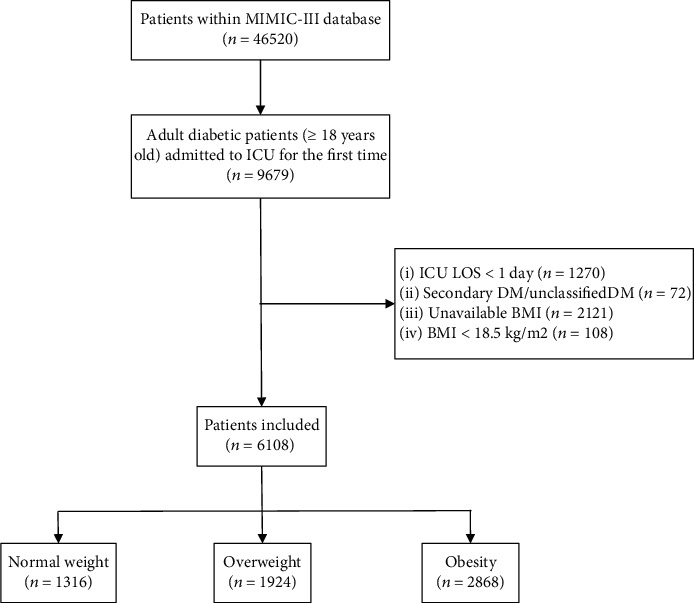
Flowchart of study cohort selection. MIMIC-III: Medical Information Mart for Intensive Care III; ICU: intensive care unit; LOS: length of stay; DM: diabetic mellitus; BMI: body mass index.

Figure 1 is a flowchart of study cohort selection. After excluding 3571 patients, a total of 6108 eligible patients were eventually enrolled in our study and were divided into three groups: the normal weight group (*n* = 1316), overweight group (*n* = 1924), and obesity group (*n* = 2868). The baseline characteristics of these patients were listed in Table 1. Compared with normal weight patients, obese patients tended to be younger (70.8 ± 14.2 vs. 65.2 ± 12.2), female (39.2% vs. 43.5%), white (64.4% vs. 69.0%), and with a history of type 2 DM (T2DM) (87.3% vs. 94.7%). The proportion of patients with elective admission in the obesity group (20.1%) and overweight group (19.0%) was higher than that in the normal weight group (14.4%). Moreover, obese and overweight patients were more likely to be admitted to cardiac surgery recovery unit (CSRU) (32.7% vs. 35.4% vs. 28.7%) and have a lower APS (41.0 (32.0, 56.0) vs. 42.0 (32.0, 55.5) vs. 45.0 (35.0, 60.0)) compared with normal weight patients. There was no significant difference (*P* > 0.05, respectively) in the prevalence of the included comorbidities between the normal weight group and obesity group, except for AMI (20.8% vs. 17.5%, *P* = 0.01) and malignancy (4.2% vs. 2.2%, *P* < 0.001).

### 3.2. Clinical Outcomes

The primary and secondary outcomes are presented in Table 2. A total of 665 (10.9%) patients died within 30 days after ICU admission and 962 (15.7%) patients died within 90 days. The 30-day, 90-day, ICU, and hospital mortality in the normal weight group were approximately 1.8 times and 1.5 times higher than those in the obesity group and overweight group, respectively (*P* < 0.001, respectively). At the same time, the ICU (2.9 (1.7, 5.3) vs. 2.7 (1.6, 4.8) vs. 2.8 (1.8, 5.0)) and hospital LOS (8.3 (5.4, 14.0) vs. 7.9 (5.1, 13.0) vs. 8.3 (5.3, 13.6)) in the obesity group and overweight group were not longer than those in the normal weight group. Compared with normal weight patients, obese patients had significantly higher incidence of mechanical ventilation (58.8% vs. 64.7%, *P* < 0.001) but no longer ventilation duration (19.3 (7.0, 73.1) vs. 19.0 (6.0, 93.7), *P* = 1). The requirements for RRT and vasopressor therapy during the ICU stay were similar among the three groups.

### 3.3. Association between BMI and Mortality

**Figure 2:**
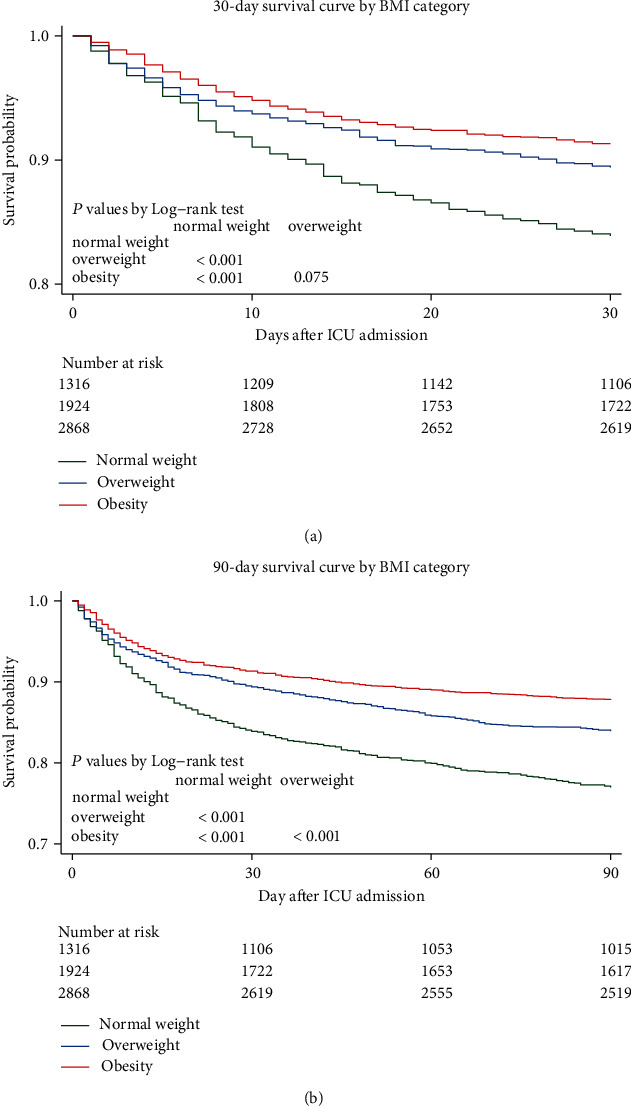
Kaplan-Meier curves of 30-day (a) and 90-day (b) mortality by BMI category. The numbers at risk refer to the patients' number at the beginning of the time periods. BMI: body mass index; ICU: intensive care unit.

Kaplan-Meier survival curves at 30 days and 90 days after ICU admission are shown in Figure 2, indicating the notable survival advantages in the obesity group and overweight group compared with the normal weight group (*P* < 0.001, respectively). However, obese patients did not show significant survival advantage within 30 days after admission to ICU, compared with their overweight counterparts (*P* = 0.075).

Table 3 summarizes the results from Cox regression. We detected significant protective effects of obesity and overweight in comparison to normal weight on 30-day mortality. The unadjusted HRs (95% CIs) of obesity and overweight were 0.52 (0.43, 0.63) and 0.64 (0.53, 0.78), respectively, compared with the reference of normal weight. When adjusted for age, gender, and ethnicity in model-1, the HRs (95% CIs) of obesity and overweight were 0.63 (0.53, 0.77) and 0.69 (0.57, 0.83), respectively. With further adjustment for the confounders in model-2, overweight patients had a significant 0.76-fold (95% CI 0.62, 0.92, *P* = 0.006) risk of dying and obese patients had an even lower risk (HR 0.62; 95% CI 0.51, 0.75, *P* < 0.001) compared with normal weight patients. The similar protective effects of obesity and overweight were also observed for 90-day mortality.

### 3.4. Post Hoc Analyses

Subgroup analyses revealed the association between BMI category and 30-day and 90-day mortality of patients with different baseline characteristics, as shown in Table 4. Obesity and overweight were independently associated with the decreased risks of 30-day and 90-day death in patients with emergency admission, but the association could not be observed in those with elective and urgent admission. Both obese patients admitted to medical intensive care unit (MICU) and overweight patients admitted to the coronary care unit (CCU) had significant lower risks of 30-day (*P* = 0.039 and *P* = 0.011, respectively) and 90-day (*P* = 0.001 and *P* = 0.004, respectively) death. For patients with respiratory failure, heart failure, or malignancy, obesity rather than overweight was associated with the lower risks of 30-day and 90-day death. There were protective effects of obesity and overweight on patients with and without AMI. However, the protective effects were not observed in patients with type 1 DM (T1DM) within 30 or 90 days after ICU admission.

**Figure 3:**
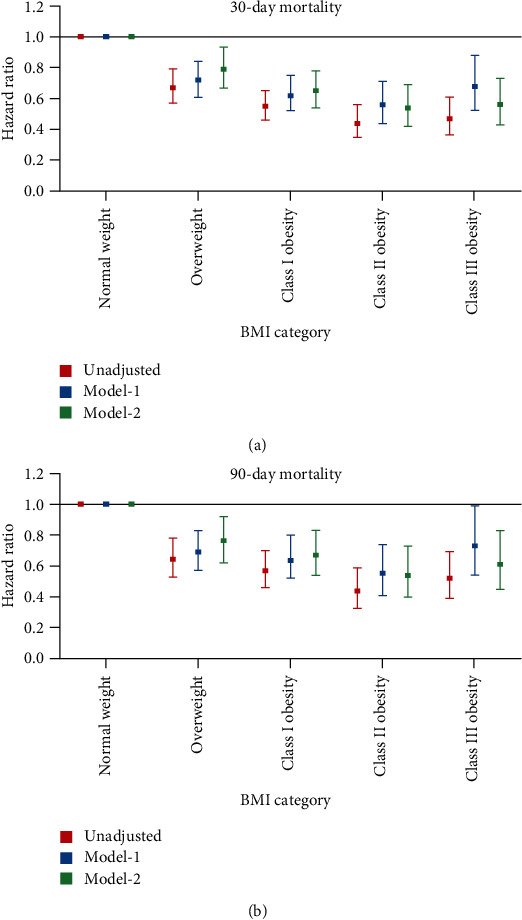
Unadjusted, model-1 and model-2 hazard ratios of 30-day (a) and 90-day (b) mortality in the five groups divided according to BMI. Normal weight category acts as the reference group. The confounder adjustment in model-1 and model-2 is the same as that in Table 3. BMI: body mass index.

To investigate the prognosis of ICU diabetic patients with different degrees of obesity, the included patients were regrouped into five groups according to BMI and then three cox regression models were reconstructed. As shown in Figure 3, all obese patients, including those with morbid obesity, had lower risks of 30-day and 90-day death compared with normal weight patients, of which class II obese patients had the lowest risks. In model-2, class II obese patients had a 0.54-fold (95% CI 0.40, 0.73) risk of 30-day death and a 0.53-fold (95% CI 0.42, 0.69) risk of 90-day death while morbidly obese patients had a 0.61-fold (95% CI 0.44, 0.83) risk of 30-day death and a 0.54-fold (95% CI 0.43, 0.73) risk of 90-day death, compared with those with normal weight.

## 4. Discussion

In this large, single-center, retrospective cohort study, we found that obesity and overweight diabetic patients admitted to the ICU had lower risks of 30-day and 90-day death compared with those of normal weight. Meanwhile, there were no increases in ICU and hospital LOS in the obesity group and overweight group. In comparison with normal weight diabetic patients, obese diabetic patients were more likely to receive mechanical ventilation but did not have significantly longer ventilation duration.

According to the WHO definition of obesity, the prevalence of obesity in the present study was 46.1%, which was higher than that in the previous studies [10–21]. It was probably due to the only inclusion of diabetic patients in our study. We also noted that obese individuals accounted for 48.3% of patients with T2DM while 31.4% of patients with T1DM were obese. These were in alignment with the previous investigation [30–33], suggesting that obesity is common in diabetic population, including patients with T2DM and T1DM.

Patients with higher BMI categories have been found to have greater incidences of respiratory failure and mechanical ventilation [16]. In our study, there was no significant difference (*P* = 0.248) in the prevalence of respiratory failure between the obesity group and normal weight group, but patients in the obesity group were more likely to receive mechanical ventilation. Although the overweight group had a lower prevalence of respiratory failure compared with the normal weight group, a smaller incidence of mechanical ventilation was not seen. We speculate that physicians in BIDMC were more vigilant about obese and overweight diabetic patients, perhaps opting for mechanical ventilation electively more often because of the concerns regarding airway management and earlier aggressive care. Additionally, unlike some previous findings [9, 16, 17, 34], our study showed that the ventilation duration in the obesity group was not longer than that in the normal weight group. This observation was unusual, as obese patients always have reduced compliance of the respiratory system and increased work of breathing and abdominal pressure, resulting in increased risks of atelectasis, aspiration, and pneumonia, and would therefore be expected to have longer ventilation duration [8, 35]. It might be due to the fact that obese diabetic patients received more attention and were triaged to higher standards of care, thus reducing the incidence of ventilator-related complications and ultimately avoiding prolonged mechanical ventilation. In fact, we were not the only ones to observe this result [14, 18].

As others have noted [10, 13, 14], we found that obese and overweight patients tended to be younger and have lower illness severity scores compared with those of normal weight. We also observed that the proportion of patients with elective admission in the obesity group and overweight group was higher than that in the normal weight group. These differences might partly reflect intrinsic difference in the general health and explain our results. However, even with adjustment for the important confounders in this study, obesity and overweight were still associated with lower risk of dying, indicating their notable survival advantages compared with normal weight. Similar results have been shown in several previous studies [11–13]. In contrast, the studies of Bochicchio et al. [17] and Bercault et al. [20] have suggested that obesity was an independent risk factor for death in critically ill patients. The discrepancies among these results might be caused by the differences in patient populations, BMI classification criteria, and research methods.

Unexpectedly, when we regrouped the included patients into five groups and reconstructed the Cox regression models, we found that even morbidly obese patients had survival advantage over normal weight patients. It has never been seen in the previous studies [10, 12–14, 34, 36], including the study of Druml et al. [13], which showed that morbid obesity was not associated with the risk of dying in critically ill patients with DM. According to the available information, the relationship between morbid obesity and the prognosis of critically ill diabetic patients is not completely determined and should be further investigated.

In another post hoc analysis, we found that the beneficial effects of obesity and overweight existed in most subgroups. Interestingly, in patients with T1DM, obesity and overweight were not associated with lower risk of dying. Limited by the small number of patients with T1DM in our study, this result should be treated with caution. Moreover, Druml et al. [13] found that a U-shaped relationship between BMI and hospital mortality was indicated in diabetic patients who did not require insulin therapy ahead of ICU admission but not in those who required insulin therapy. However, we could not verify this finding in the present study, due to the lack of information about patients' insulin use ahead of ICU admission.

There are several reasons that may explain our findings—obesity and overweight were associated with greater survival in critically ill diabetic patients. First, adipokines and inflammatory mediators (e.g., leptin and adiponectin) released by adipocytes could mitigate the deleterious inflammatory response and thus improve host survival in response to critical illness [37]. Second, abundant adipose tissue in obese and overweight patients could provide energy and lipid soluble nutrients necessary to sustain organ function during the extremely acute catabolic state [38]. Third, obese and overweight diabetic patients may have a lower threshold for ICU admission compared with their normal weight counterparts, meaning the disease severity is less than expected. As our study showed, these patients had a lower APS. Fourth, obese diabetic patients are more likely to receive more attention from medical staff and higher standards of care in comparison with normal weight patients, due to the early reports of decreased survival in obese patients [17, 20].

To our knowledge, the present study was the first study to specially and systematically assess the effect of obesity and overweight on the outcomes among critically ill diabetic patients. The study included a relatively large number of participants, improving the reliability of the findings. However, several limitations should be acknowledged. First, it was a single-center, retrospective study. Hence, our findings might not be generalizable to other centers and a causal relationship between obesity and ICU outcomes could not be inferred. Second, we could not discount the inaccuracy of body weight and height data. These variables are often estimated rather than measured in ICU, and estimates could be inaccurate [39]. Third, body weight measured within 24 hours of ICU admission might be significantly different from a patients' actual body weight because of volume overload or depletion. This limitation might only lead to misclassification of few patients in the different BMI categories and therefore might not change our conclusions. Fourth, the single measurement of BMI lacked the ability to differentiate between lean, fat mass, and the distribution of fat [40]. The simultaneous measurement of other anthropometric indicators such as waist circumference and body fat percentage would allow more comprehensive analyses, but these indicators were not recorded in the MIMIC-III database. Lastly, some potential confounders such as insulin use prior to ICU admission [13] and nutritional intake during the ICU stay [41] were not available from the database, which might affect the reliability of the results.

## 5. Conclusions

Our study demonstrated that obesity and overweight were independently associated with greater survival in critically ill diabetic patients, without increasing the ICU and hospital LOS. Large multicenter prospective studies are needed to confirm our findings, and the underlying mechanisms warrant further investigation.

## Figures and Tables

**Table 1:** Baseline characteristics of the study patients by BMI category.

Characteristics	Overall (*n* = 6108)	Normal weight (*n* = 1316)	Overweight (*n* = 1924)	Obesity (*n* = 2868)	*P* value

BMI (kg/m^2^)	30.9 ± 8.0	22.7 ± 1.6	27.4 ± 1.4^a^	37.1 ± 7.4^a^	<0.001
Age (years)	67.7 ± 13.0	70.8 ± 14.2	69.1 ± 12.7^a^	65.2 ± 12.2^a^	<0.001
Gender, male, *n* (%)	3680 (60.2)	800 (60.8)	1260 (65.5) ^c^	1620 (56.5) ^c^	<0.001
Ethnicity, *n* (%)					<0.001
White	4134 (67.7)	848 (64.4)	1308 (68.0)	1833 (69.0)^b^	0.014
Black	618 (10.1)	136 (10.3)	167 (8.7)	280 (11.0)	0.033
Other	507 (8.3)	145 (11.0)	168 (8.7)	188 (6.8)^a^	<0.001
Unknown	849 (13.9)	187 (14.2)	281 (14.6)	352 (13.3)	0.404
Admission type, *n* (%)					<0.001
Emergency	4783 (78.3)	1085 (82.4)	1487 (77.3)^a^	2211 (77.1)^a^	<0.001
Elective	1131 (18.5)	190 (14.4)	365 (19.0)^b^	576 (20.1)^a^	<0.001
Urgent	194 (3.1)	41 (3.1)	72 (3.7)	81 (2.8)	0.204
ICU type, *n* (%)					<0.001
MICU	1810 (29.6)	420 (31.9)	497 (25.8) ^a^	893 (31.1)	<0.001
CCU	1173 (19.2)	252 (19.1)	399 (20.7)	522 (18.2)	0.092
CSRU	1997 (32.7)	378 (28.7)	682 (35.4)^a^	937 (32.7)^c^	<0.001
SICU	734 (12.0)	180 (13.7)	221 (11.5)	333 (11.6)	0.111
TSICU	394 (6.5)	86 (6.5)	125 (6.5)	183 (6.4)	0.977
DM, T2DM, *n* (%)	5620 (92.0)	1149 (87.3)	1756 (91.3)^a^	2715 (94.7)^a^	<0.001
Scoring systems^∗^					
SOFA	4.0 (3.0, 6.0)	4.0 (3.0, 6.0)	4.0 (3.0, 6.0)	4.0 (3.0, 6.0)	0.752
APS III	43.0 (33.0, 57.0)	45.0 (35.0, 60.0)	42.0 (32.0, 55.5)^a^	41.0 (32.0, 56.0)^a^	<0.001
Comorbidity, *n* (%)					
AMI	1208 (19.8)	274 (20.8)	432 (22.5)	502 (17.5)^c^	<0.001
Stroke	162 (2.7)	46 (3.5)	38 (2.0)^c^	78 (2.7)	0.029
Renal failure	2075 (34.0)	442 (33.6)	628 (32.6)	1005 (35.0)	0.215
Respiratory failure	1120 (18.3)	246 (18.7)	294 (15.3)^c^	580 (20.2)	<0.001
Heart failure	2370 (38.8)	495 (37.6)	735 (38.2)	1140 (39.7)	0.340
Malignancy	183 (3.0)	55 (4.2)	64 (3.3)	64 (2.2)^a^	0.002

Abbreviations: BMI: body mass index; ICU: intensive care unit; MICU: medical intensive care unit; CCU: coronary care unit; CSRU: cardiac surgery recovery unit; SICU: surgical intensive care unit; TSICU: thoracic surgery intensive care unit; DM: diabetic mellitus; SOFA: sequential organ failure assessment; APS III: acute physiology score III; AMI: acute myocardial infarction. Normally distributed data are presented as mean ± SD, nonnormally distributed data are presented as median (IQR), and categorical variables are presented as *n* (%). ^∗^Scores were calculated within 24 h of ICU admission. ^a^*P* < 0.001 compared with the normal weight group. ^b^*P* < 0.01 compared with the normal weight group. ^c^*P* < 0.05 compared with the normal weight group.

**Table 2:** Clinical outcomes of the study patients by BMI category.

Outcomes	Overall (*n* = 6108)	Normal weight (*n* = 1316)	Overweight (*n* = 1924)	Obesity (*n* = 2868)	*P* value

Primary					
30-day mortality, *n* (%)	665 (10.9)	212 (16.1)	204 (10.6)^a^	249 (8.7)^a^	<0.001
90-day mortality, *n* (%)	962 (15.7)	303 (23.0)	309 (16.1)^a^	350 (12.2)^a^	<0.001
Secondary					
ICU mortality, *n* (%)	394 (6.5)	122 (9.3)	115 (6.0)^a^	157 (5.5)^a^	<0.001
Hospital mortality, *n* (%)	547 (9.0)	177 (13.4)	158 (8.2)^a^	212 (7.4)^a^	<0.001
ICU LOS (days)	2.8 (1.7, 5.1)	2.8 (1.8, 5.0)	2.7 (1.6, 4.8)	2.9 (1.7, 5.3)	0.012
Hospital LOS (days)	8.2 (5.3, 13.6)	8.3 (5.3, 13.6)	7.9 (5.1, 13.0)	8.3 (5.4, 14.0)	0.012
Ventilation, *n* (%)	3823 (62.6)	774 (58.8)	1192 (62.0)	1857 (64.7)^a^	0.001
Ventilation duration (hours)	18.0 (6.0, 75.2)	19.3 (7.0, 73.1)	15.1 (5.2, 55.8)^b^	19.0 (6.0, 93.7)	<0.001
RRT, *n* (%)	506 (8.3)	118 (9.0)	164 (8.5)	224 (7.8)	0.407
Vasopressor, *n* (%)	1574 (25.8)	341 (25.9)	498 (25.9)	735 (25.6)	0.972

Abbreviations: BMI: body mass index; ICU: intensive care unit; LOS: length of stay; RRT: renal replacement therapy. Nonnormally distributed data are presented as median (IQR), and categorical variables are presented as *n* (%). ^a^*P* < 0.001 compared with the normal weight group. ^b^*P* < 0.05 compared with the normal weight group.

**Table 3:** HRs (95%CIs) of 30-day and 90-day mortality according to BMI category.

	Unadjusted		Model-1^†^		Model-2^‡^	
	HR (95% CI)	*P* value	HR (95% CI)	*P* value	HR (95% CI)	*P* value

30-day mortality						
Normal weight^∗^	1.00		1.00		1.00	
Overweight	0.64 (0.53, 0.78)	<0.001	0.69 (0.57, 0.83)	<0.001	0.76 (0.62, 0.92)	0.006
Obesity	0.52 (0.43, 0.63)	<0.001	0.63 (0.53, 0.77)	<0.001	0.62 (0.51, 0.75)	<0.001
90-day mortality						
Normal weight^∗^	1.00		1.00		1.00	
Overweight	0.67 (0.57, 0.79)	<0.001	0.72 (0.61, 0.84)	<0.001	0.79 (0.67, 0.93)	0.005
Obesity	0.50 (0.43, 0.58)	<0.001	0.62 (0.53, 0.72)	<0.001	0.60 (0.51, 0.70)	<0.001

Abbreviations: HR: hazard ratio; CI: confidence interval; BMI: body mass index. ^∗^Normal weight category acts as the reference group. ^†^Model-1 was adjusted for the confounders age, gender, and ethnicity. ^‡^Model-2 was adjusted for the confounders age, gender, ethnicity, admission type, ICU type, DM, SOFA, APS III, AMI, stroke, renal failure, respiratory failure, heart failure, and malignancy. In 30-day mortality model, SOFA, APS III, renal failure, heart failure, and malignancy were entered as time-dependent covariates. In 90-day mortality model, SOFA, APS III, renal failure, respiratory failure, heart failure, and malignancy were entered as time-dependent covariates.

**Table 4:** Adjusted HRs (95%CIs)^†^ of 30-day and 90-day mortality by BMI category within different subgroups.

Subgroups	*N*	BMI category of 30-day mortality			BMI category of 90-day mortality		
		Normal weight^∗^	Overweight	Obesity	Normal weight^∗^	Overweight	Obesity

Admission type							
Emergency	4783	1.00	0.71 (0.58, 0.86)	0.67 (0.55, 0.82)	1.00	0.75 (0.63, 0.88)	0.65 (0.55, 0.77)
Elective	1131	1.00	0.64 (0.26, 1.55)	0.51 (0.21, 1.25)	1.00	0.65 (0.32, 1.32)	0.51 (0.25, 1.05)
Urgent	194	1.00	1.15 (0.37, 3.62)	0.62 (0.15, 2.54)	1.00	0.76 (0.31, 1.86)	0.50 (0.18, 1.40)
ICU type							
MICU	1810	1.00	1.03 (0.77, 1.38)	0.74 (0.56, 0.98)	1.00	0.95 (0.75, 1.22)	0.66 (0.52, 0.84)
CCU	1173	1.00	0.58 (0.38, 0.89)	0.70 (0.46, 1.05)	1.00	0.59 (0.42, 0.84)	0.72 (0.51, 1.01)
CSRU	1997	1.00	0.71 (0.34, 1.47)	1.18 (0.62, 2.28)	1.00	0.65 (0.39, 1.08)	0.82 (0.51, 1.34)
SICU	734	1.00	0.53 (0.33, 0.85)	0.34 (0.21, 0.57)	1.00	0.72 (0.49, 1.05)	0.39 (0.26, 0.59)
TSICU	394	1.00	0.48 (0.24, 0.96)	0.45 (0.22, 0.91)	1.00	0.72 (0.41, 1.27)	0.53 (0.28, 0.97)
DM							
T1DM	488	1.00	0.44 (0.19, 1.02)	0.43 (0.17, 1.06)	1.00	0.64 (0.33, 1.27)	0.49 (0.22, 1.05)
T2DM	5620	1.00	0.69 (0.57, 0.85)	0.63 (0.52, 0.77)	1.00	0.71 (0.61, 0.84)	0.61 (0.52, 0.72)
AMI							
No	4900	1.00	0.73 (0.58, 0.91)	0.65 (0.52, 0.80)	1.00	0.79 (0.66, 0.95)	0.63 (0.52, 0.75)
Yes	1208	1.00	0.57 (0.38, 0.86)	0.60 (0.40, 0.89)	1.00	0.52 (0.37, 0.73)	0.60 (0.43, 0.84)
Stroke							
No	5946	1.00	0.69 (0.57, 0.85)	0.65 (0.54, 0.79)	1.00	0.73 (0.62, 0.86)	0.63 (0.52, 0.75)
Yes	162	1.00	0.74 (0.31, 1.76)	0.43 (0.18, 1.05)	1.00	0.83 (0.40, 1.71)	0.46 (0.22, 0.98)
Renal failure							
No	4033	1.00	0.60 (0.45, 0.80)	0.47 (0.35, 0.64)	1.00	0.59 (0.47, 0.75)	0.45 (0.35, 0.57)
Yes	2075	1.00	0.75 (0.58, 0.97)	0.71 (0.55, 0.90)	1.00	0.81 (0.65,1.00)	0.70 (0.57, 0.86)
Respiratory failure							
No	4988	1.00	0.68 (0.53, 0.88)	0.57 (0.44, 0.73)	1.00	0.69 (0.56, 0.84)	0.55 (0.44, 0.67)
Yes	1120	1.00	0.82 (0.61, 1.11)	0.66 (0.50, 0.88)	1.00	0.92 (0.70, 1.19)	0.67 (0.52, 0.86)
Heart failure							
No	3738	1.00	0.56 (0.42, 0.73)	0.57 (0.44, 0.73)	1.00	0.61 (0.49, 0.76)	0.57 (0.45, 0.71)
Yes	2370	1.00	0.85 (0.64, 1.12)	0.70 (0.53, 0.92)	1.00	0.85 (0.68, 1.06)	0.65 (0.52, 0.82)
Malignancy							
No	5925	1.00	0.69 (0.56, 0.85)	0.68 (0.56, 0.83)	1.00	0.73 (0.62, 0.87)	0.66 (0.56, 0.78)
Yes	183	1.00	0.81 (0.46, 1.42)	0.48 (0.25, 0.91)	1.00	0.71 (0.44, 1.16)	0.46 (0.27, 0.78)

Abbreviations: HR: hazard ratio; CI: confidence interval; BMI: body mass index; ICU: intensive care unit; MICU: medical intensive care unit; CCU: coronary care unit; CSRU: cardiac surgery recovery unit; SICU: surgical intensive care unit; TSICU: thoracic surgery intensive care unit; DM: diabetic mellitus; AMI: acute myocardial infarction. ^†^HRs were adjusted for age, gender, and ethnicity. ^∗^Normal weight category acts as the reference group.

## Data Availability

The data that support the findings of this study are available from the MIMIC-III database but restrictions apply to the availability of these data, which were used under license for the current study, and so are not publicly available. Data are however available from the authors upon reasonable request and with permission of the MIMIC-III database.
